# Synchronous large tumoral pseudoangiomatous stromal hyperplasia (PASH) in the breast and axilla with subsequent carcinoma in the contralateral breast: routine and strain imaging with histopathological correlation

**DOI:** 10.1259/bjrcr.20150017

**Published:** 2015-07-27

**Authors:** T R Shimpi, V Baksa Reynolds, S Shikhare, S Srinivasan, M J Clarke, W C G Peh

**Affiliations:** Department of Diagnostic Radiology, Khoo Teck Puat Hospital, Alexandra Health, Singapore

## Abstract

Pseudoangiomatous stromal hyperplasia (PASH) is a benign breast disorder with the tumoral variety being extremely rare. We report a rare case of synchronous, massive axillary and breast tumoral PASH in a 55-year-old post-menopausal woman. Mammography, ultrasonography and sonoelastography features are illustrated with histopathological correlation. A high-grade invasive ductal carcinoma was detected in the contralateral breast on annual follow-up imaging. Radiologists need to be familiar with the imaging appearances of PASH and be aware of its association with subsequent cancer risk. To the best of our knowledge, the present case of synchronous tumoral PASH in the breast and axillary tissue is the second reported case till now.

## Summary

Pseudoangiomatous stromal hyperplasia (PASH) was first reported in 1986 with increased detection owing to increasing core biopsies. Massive nodular PASH in the breast and simultaneous axillary tumoral PASH is extremely rare, with only one such case having been described in literature until now. The sonoelastographic features of tumoral PASH have not been previously described.

We present the imaging features, including the sonoelastographic findings, of such a rare case of co-existing tumoral PASH in a post-menopausal patient who after mastectomy developed high-grade invasive ductal carcinoma in the contralateral breast.

## Clinical presentation

A 55-year-old post-menopausal female presented with painless enlargement of the left breast and axilla for over 3 months. On clinical examination, her left breast was enlarged with a soft, mobile lump palpable in the periareolar region. The overlying skin was thickened and erythematous, with retraction of the nipple. A soft lump was also palpable in the left axilla. She had systemic morbidities, such as chronic renal disease and ischaemic heart disease.

## Differential diagnosis

The possibilities based on the clinical picture included:

1. breast neoplasm with axillary lymphadenopathy
2. breast and axillary benign masses
3. breast abscess/collection.

## Investigations/imaging findings

**Figure 1.**
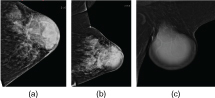
Mammogram, left craniocaudal and mediolateral oblique view, showing a large, well-circumscribed mass involving the left central breast and upper lateral quadrant with associated skin thickening and retraction of the nipple (a,b). Extended axillary mammogram showing a well-defined, smooth, dense mass occupying the whole axilla (c).

Routine mammogram revealed a large, well-circumscribed, homogeneously dense mass of 6 cm in the left central and upper lateral quadrant with associated skin thickening and retraction of the nipple (Figure 1a,b). The extended axillary mammogram showed a well-defined, smooth, dense mass occupying the whole axilla (Figure 1c).

**Figure 2.**
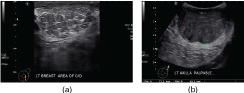
Ultrasonographic image showing a partially circumscribed, oval heterogeneously hypoechoic mass in the left breast with echogenic striations (a). Ultrasonographic image of the left axilla showing a 6 × 5 × 2 cm, well-defined, extremely hypoechoic, slightly inhomogeneous mass occupying the entire left axilla (b).

**Figure 3.**
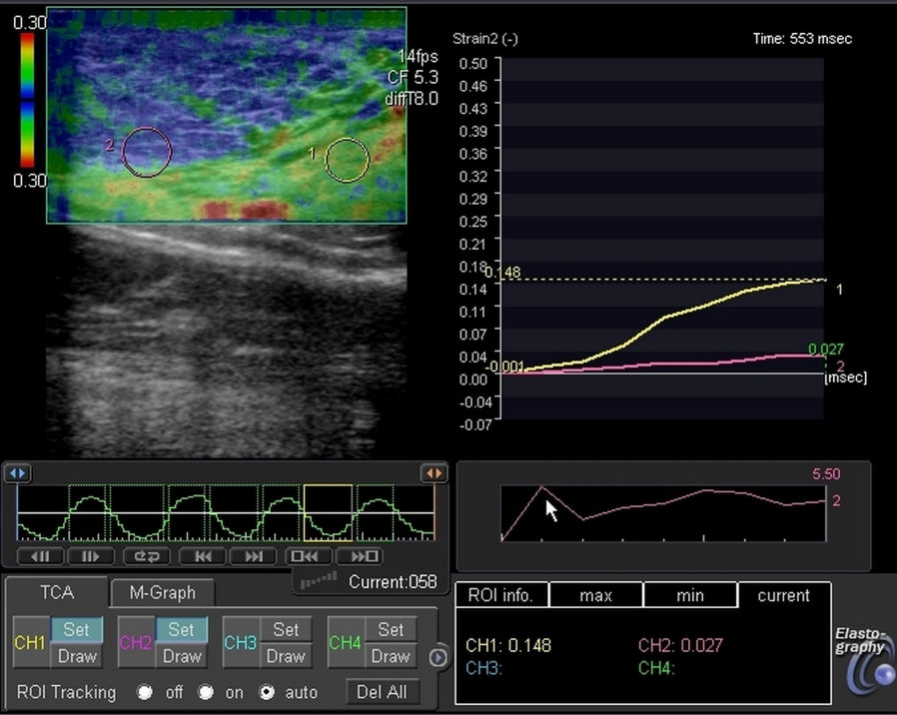
Sonoelastographic image showing the strain ratio of both the lesions and the surrounding tissue at several foci. The strain values range between 2.5 and 5.2, equivalent to the surrounding breast tissue, indicative of benign aetiology.

**Figure 4.**
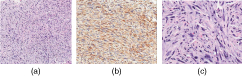
Histopathological evaluation. The cores showed predominant stroma with pseudoangiomatous stromal hyperplasia and few cystically dilated mammary ducts (a–c).

On ultrasonography, the left breast mass was a well-circumscribed, oval, hypoechoic lesion measuring approximately 7 × 6 × 5 cm in size, parallel to the skin and showing increased posterior transmission. Ultrasonography of the left axilla showed a well-defined, hypoechoic, slightly inhomogeneous mass, extending to the skin surface and displacing the axillary tissue and vessels, measuring about 6 × 5 × 2 cm with increased posterior transmission (Figure 2a,b). On sonoelastographic studies, the strain ratio between the breast and axillary lesions and the surrounding tissue, measured at several foci, showed the strain values ranging between 2.5 and 3, equal to the surrounding tissue, indicating a benign aetiology (Figure 3). Histopathological evaluation of the breast and axillary masses revealed ducts exhibiting the usual type hyperplasia surrounded by the stroma showing PASH (Figure 4a–c).

## Treatment

The patient subsequently underwent left mastectomy and axillary lumpectomy.

## Outcome and follow-up

**Figure 5.**
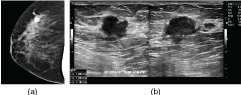
Annual follow-up mammogram. Craniocaudal view of the contralateral breast showing an ill-defined, spiculated density with microcalcifications in the right central breast (a). Ultrasonographic image reveals an irregular, lobulated, hypoechoic mass in the right breast at 10 o'clock position (b).

After left mastectomy, annual follow-up mammogram of the contralateral right breast revealed an ill-defined, spiculated mass with microcalcifications (Figure 5a). The mass measured approximately 1.8 × 1.5 cm. On ultrasound, it appeared as a lobulated, hypoechoic mass (Figure 5b) with raised vascularity on colour Doppler with an associated abnormal axillary lymph node. It was histologically proven to be an invasive ductal carcinoma (grade 3).

## Discussion

Although first described in 1986 with well-documented pathological features, there is little in the radiology literature describing the imaging features of PASH. It is important to be aware of the radiological findings of PASH as it is identified in as many as 25% of biopsy specimens. PASH is a benign breast disorder characterized by dense proliferation of the breast stroma forming slit-like pseudovascular spaces lined by spindle cells. PASH is often accompanied by benign or malignant breast lesions. It is rare for PASH to form a palpable nodule. The nodular and tumoral variety of PASH is extremely rare and it is important to differentiate this entity from low-grade angiosarcoma. Microscopic appearance of PASH is similar to endothelial spindle cells forming vessel-like slits within the stroma, which are not true vessels covered with endothelia but rather vacant spaces bordered by myofibroblasts. Some authors recommend wide local excision of PASH, as it recurs after excision. Powel et al^1^ reported a case in which, owing to multiple recurrences, bilateral mastectomy was performed.

PASH occurs in pre- or post-menopausal females receiving hormone replacement therapy. The well-accepted hypothesis is that the stromal hyperplasia in PASH results from an exaggerated responsiveness of myofibroblasts to hormonal stimuli. The main hormone implicated is progesterone. Our case was thus distinct as the patient was postmenopausal and had not received hormonal replacement therapy. Rapid growth in size is a feature pertinent to tumoral PASH. It can occur in extramammary sites such as the axilla, with very few cases having been reported until now.^2,3^ The simultaneous occurrence of PASH in the breast and the axilla is also extremely rare, with only one such case reported until now by Jordan et al.^3^

The mammographic and sonographic features of PASH can be quite non-specific and can show a wide range of spectrum. On mammography, PASH can appear as a partially/well-circumscribed mass or an asymmetric density. PASH can occur in the middle of a surgical or radial scar, ductal carcinoma *in situ*, atypical ductal hyperplasia or fibrocystic change, appearing as an architectural distortion or calcifications. In one study by Virk et al,^4^ PASH was detected in only 31 of 90 patients on mammography, indicating that some cases are not detected mammographically.

On ultrasonography, it exhibits variable findings, ranging from well to ill-defined, homogeneous to inhomogeneous with varying posterior enhancement, sometimes mimicking other benign lesions.^5^ Few large nodular PASH lesions may reveal numerous reticular areas with scattered cystic changes. Sometimes, co-existing fibrocystic changes make the lesion more heterogeneous. The sonoelastographic features pertinent to tumoral PASH are not well described in the literature.

MRI appearance has been described in very few cases.^4^ According to two reports that used a dedicated breast coil, PASH was isointense on *T*_1_ weighted images and showed a hyperintense linear reticular “lace-like” pattern on *T*_2_ weighted images. The presence of these linear reticular lines may potentially be a helpful feature in diagnosing PASH. These lesions revealed homogeneous enhancement after contrast. The mean kinetic curve with persistent enhancement was noted in these reports.^5^

On histopathological analysis, PASH exhibits usual type hyperplasia of the affected ducts, epithelial and papillomatous proliferation, cystic dilatation of the ducts and vascular endothelial-like spindle cells, forming vessel-like slits in the stroma.

There is a possibility of co-existing carcinoma, that is, a small portion of PASH in the vicinity of carcinoma. Two relatively large studies showed that 4% and 10% of total cases had co-existing carcinoma at the site of PASH.^2,4^ Histological diagnosis is therefore necessary to exclude malignancy. In a study by Hargaden et al,^2^ 2 of 149 patients had subsequent diagnosis of malignancy in the opposite breast. The subsequent development of carcinoma at the site of PASH is also known.^6^

Thus, PASH is a rare benign lesion that can have variable imaging features and may mimic malignant or other benign conditions. It is usually characterized by rapid growth and recurrence after excision. Tumoral PASH is known to have the potential for malignant transformation and subsequent development of contralateral cancer after mastectomy.^6^

## Learning points

1. Radiologists need to consider tumoral PASH as one of the differential diagnosis of well-circumscribed, rapidly growing breast lesions.
2. Knowledge of its association with subsequent cancer risk is helpful to the clinicians and radiologists alike, in view of regular follow-up with mammography and ultrasound for early detection of subsequent malignant growth at the site of PASH or in the contralateral breast.
3. PASH is usually characterized by recurrence after excision.
